# The miR-200 Family in Non-Small-Cell Lung Cancer: Molecular Mechanisms, Clinical Applications, and Therapeutic Implications

**DOI:** 10.3390/genes16111312

**Published:** 2025-11-02

**Authors:** Nobuaki Kobayashi, Yukihito Kajita, Fangfei Yang, Nobuhiko Fukuda, Kohei Somekawa, Ayami Kaneko, Seigo Katakura

**Affiliations:** 1 Department of Pulmonology, Yokohama City University Graduate School of Medicine, 3-9 Fukuura, Kanazawa-ku, Yokohama 236-0004, Japan; 2 Department of Thoracic Oncology, Kanagawa Cancer Center, 2-3-2 Nakao, Asahi-ku, Yokohama 241-8515, Japan

**Keywords:** non-small cell lung cancer (NSCLC), miR-200, epithelial-mesenchymal transition (EMT), biomarker, therapeutic resistance

## Abstract

Non-small-cell lung cancer (NSCLC) remains a leading cause of cancer-related mortality worldwide, demanding improved biomarkers and therapeutic approaches. This review synthesizes the extensive evidence positioning the miR-200 family as a master regulator of NSCLC progression. We detail the core molecular circuitry centered on the bistable, double-negative feedback loop between miR-200 and the ZEB1/ZEB2 transcription factors, which governs epithelial–mesenchymal transition (EMT). This review connects this central mechanism to critical clinical challenges, including the development of resistance to EGFR-targeted therapies and the regulation of immune evasion through PD-L1 expression and CD8+ T cell infiltration. We evaluate the strong clinical evidence for the miR-200 family’s utility as a diagnostic, prognostic, and predictive biomarker. Finally, we explore emerging therapeutic strategies that target this network, including miRNA replacement, epigenetic reactivation, and rational combinations with immunotherapy and targeted agents. We synthesize evidence positioning the miR-200/ZEB feedback circuit as a central regulatory node in NSCLC that links EMT with therapeutic resistance and immune evasion. Beyond summarizing associations, we interpret how this circuitry could inform biomarker development and rational combinations with targeted and immune therapies. Given heterogeneous study designs and non-standardized assays, translational claims remain provisional; we outline immediate priorities for assay harmonization and biomarker-stratified trials.

## 1. Introduction

Lung cancer represents a major global health burden, causing over 1.8 million deaths annually [1]. The microRNA-200 (miR-200) family has emerged as a critical regulator in non-small-cell lung cancer (NSCLC), orchestrating a molecular switch centered on a bistable feedback loop with ZEB1/ZEB2 transcription factors that governs epithelial–mesenchymal plasticity, therapeutic resistance, and immune evasion [2,3]. This regulatory network has actionable interfaces with key therapies; preclinical evidence links the miR-200c/LIN28B axis to acquired resistance to EGFR tyrosine kinase inhibitors (TKIs) [4], while the circuit’s control over PD-L1 expression modulates the tumor immune microenvironment [5]. Consequently, the miR-200 family holds significant promise as a multifaceted clinical biomarker, with evidence supporting its diagnostic, prognostic, and predictive value for both targeted therapy and immunotherapy responses [6,7,8,9].

In clinical practice, treatment decisions are guided by molecular drivers and PD-L1 expression, which are strong determinants of survival [10,11]. However, tissue-based PD-L1 assessment is limited by sample availability and spatiotemporal heterogeneity. This gap highlights the urgent need for minimally invasive surrogate biomarkers, such as circulating miR-200, to enable longitudinal monitoring and better inform therapeutic strategies.

The primary objective of this review is to synthesize the extensive evidence positioning the miR-200/ZEB axis as a central node that integrates the molecular mechanisms of both targeted therapy resistance and immune evasion in NSCLC. We aim to provide a comprehensive overview of the clinical applications of this axis as a multifaceted biomarker and to highlight emerging therapeutic strategies that target this network. Ultimately, we propose a framework for future biomarker-stratified trials designed to translate these mechanistic insights into clinical practice.

A structured literature search was conducted using the PubMed and Web of Science databases to identify relevant articles published through March 2025. This review included original research and review articles focusing on the miR-200 family in non-small-cell lung cancer (NSCLC). Conference abstracts and non-peer-reviewed articles were excluded to ensure a comprehensive and reproducible synthesis of the available evidence.

In the following sections, we first synthesise the core miR-200/ZEB circuitry that governs EMT plasticity, and then link these mechanisms to clinical phenotypes and therapeutic interfaces (EGFR-TKIs and immuno-oncology). We subsequently appraise the clinical utility of miR-200 as a biomarker and survey emerging therapeutic strategies. Finally, we outline concrete future directions to accelerate clinical translation.

## 2. The Core miR-200/ZEB Regulatory Circuit

Building on the clinical motivation introduced above, this section delineates the bistable, double-negative miR-200-ZEB1/2 loop and its epigenetic and signalling inputs that stabilise epithelial or mesenchymal states.

The miR-200 family, which comprises miR-200a, miR-200b, miR-200c, miR-141, and miR-429, orchestrates a bistable molecular switch through a double-negative feedback loop with the *ZEB1* and *ZEB2* transcription factors [12,13]. This circuit enforces mutually exclusive epithelial and mesenchymal cellular states. In the epithelial state, high levels of miR-200 suppress the translation of *ZEB1*/*ZEB2* mRNA, thereby maintaining E-cadherin expression and preserving cell–cell adhesion. Conversely, in the mesenchymal state, *ZEB1* and *ZEB2* bind to E-box elements within the miR-200 gene promoters, leading to reciprocal transcriptional repression that stabilizes the mesenchymal phenotype [14,15].

This core loop integrates a multitude of upstream signaling inputs. Positive regulators that promote an epithelial state include *p53*, which directly transactivates miR-200 expression under cellular stress, as well as the transcription factors GRHL2, KLF4, and OVOL2 [16,17]. Negative regulators that induce a mesenchymal state include the canonical EMT-inducing transcription factors SNAIL and TWIST, along with pathway-specific inputs from TGF-β (via SMAD-mediated repression), Wnt (via β-catenin/LEF complexes), and Notch signaling (via HEY1/HEY2 effectors) [18,19].

Epigenetic modifications provide an additional layer of control. In mesenchymal cells, the miR-200 promoters are silenced by CpG hypermethylation and the deposition of repressive histone marks, such as H3K27me3 and H3K9me3 [20]. These epigenetic changes create a heritable silenced state that is maintained through cell divisions.

**Figure 1:**
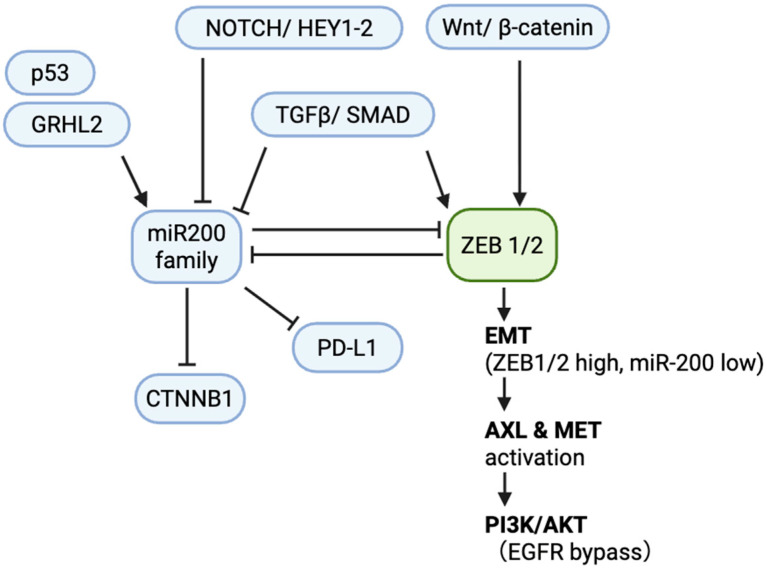
Core miR-200/ZEB circuit in NSCLC. Schematic of the double-negative feedback loop between the miR-200 family (miR-200a/b/c, miR-141, miR-429) and ZEB1/2 that regulates epithelial–mesenchymal transition (EMT). Upstream regulators include p53 and GRHL2 (activation of miR-200) and TGF-β/SMAD, Wnt/β-catenin, and Notch/HEY1-2 (repression of miR-200 and activation of ZEB1/2). Downstream targets of miR-200 include PD-L1 and CTNNB1 (β-catenin). In the mesenchymal state (ZEB1/2 high, miR-200 low), EMT is maintained with AXL/MET activation sustaining PI3K/AKT signaling, providing an EGFR-TKI bypass. Arrows denote activation; blunt lines denote repression. Abbreviations: NSCLC, non-small-cell lung cancer; EMT, epithelial–mesenchymal transition; TKI, tyrosine kinase inhibitor; EGFR, epidermal growth factor receptor; PD-L1, programmed death-ligand 1; PI3K, phosphoinositide 3-kinase; AKT, protein kinase B; TGF-β, transforming growth factor beta.

The downstream consequences of this circuit extend beyond ZEB proteins to influence a wide array of cellular functions. These include direct targeting of *CTNNB1* (modulating Wnt signaling and cell junctions), regulation of RhoE and WAVE3 (controlling actin dynamics and motility), targeting of matrix metalloproteinases and fibronectin (reducing invasive capacity), and suppression of *FLT1*/VEGFR1 (limiting angiogenic responses), a pathway also implicated in modulating EMT-driven TKI resistance [21,22,23]. Furthermore, the miR-200/ZEB axis regulates key stemness factors (SOX2, BMI1), metabolic enzymes (PKM2, LDHA), and immune modulators (PD-L1, CXCL1/2) [24,25]. The bistable and hysteretic nature of this circuit means that a significant and sustained signal is required to flip the switch between states, a dynamic that has profound implications for therapeutic resistance (Figure 1).

These circuit properties provide a mechanistic substrate for heterogeneous EMT states observed in tumours; we next examine how such states map onto disease progression and stemness features in patients.

## 3. Role of the miR-200 Family in NSCLC Phenotypes and Disease Progression

We next integrate patient-level evidence demonstrating that low miR-200 expression aligns with mesenchymal features, advanced stage, and inferior survival, while single-cell and spatial analyses contextualise this along an EMT spectrum.

Evidence from patient cohorts consistently demonstrates that low miR-200 expression in tumors correlates with mesenchymal features, advanced disease stage, and poor survival outcomes [26,27]. Large-scale studies and meta-analyses have shown that the expression levels of the miR-200 family are independent predictors of overall survival after adjusting for stage, histology, and treatment modality [7]. This prognostic significance is observed across both adenocarcinoma and squamous cell carcinoma subtypes.

Single-cell RNA sequencing has illuminated the marked intratumoral heterogeneity of miR-200 expression, revealing that tumors are composed of cellular subpopulations existing along a spectrum of EMT states rather than in binary epithelial or mesenchymal phenotypes [28]. Spatial transcriptomic analyses have further shown that miR-200-low, mesenchymal-like cells are preferentially located at the invasive fronts of tumors and within sites of lymphovascular invasion, consistent with their role in metastatic dissemination [29]. These findings help explain the frequent discordance in EMT status between primary tumors and metastatic sites.

The miR-200/EMT axis is also intricately linked to cancer stem cell (CSC) properties. Low miR-200 expression is associated with an enrichment of CD133+/ALDH1+ populations, enhanced self-renewal capacity, and resistance to anoikis [30,31]. These CSC-like features are thought to contribute to metastatic colonization and therapeutic tolerance. It is important to note, however, that this connection is context-dependent, as some studies have identified epithelial stem cell populations that maintain high miR-200 expression [32]. The clinical implication of this heterogeneity is the need for multi-site and longitudinal sampling to accurately capture the dynamic EMT state of a patient’s tumor.

Given these clinicopathologic correlations, the question becomes how EMT-linked miR-200 loss interfaces with treatment response; we therefore turn to therapeutic contexts in EGFR-targeted therapy and immuno-oncology.

## 4. Therapeutic Interfaces

This section aggregates evidence that EMT-coupled miR-200 suppression shapes both primary and acquired resistance to EGFR-TKIs and wires immune evasion through PD-L1 and the tumour microenvironment.

### 4.1. EGFR Tyrosine Kinase Inhibitors (TKIs)

Primary resistance to EGFR-TKIs in patients with activating EGFR mutations is strongly associated with baseline mesenchymal features and low miR-200 expression [33]. Complex tri-culture organoid models further validated these findings, showing that EMT-linked TKI tolerance was attenuated by either VEGF blockade (bevacizumab) or miR-200c mimic transfection. Both interventions improved TKI sensitivity and reversed EMT markers, implicating VEGF signaling in this resistance mechanism. These data nominate VEGF blockade and miR-200c restoration as rational combinatorial strategies against EMT-driven EGFR-TKI resistance [23]. The mechanisms underlying EMT-mediated resistance are multifactorial and include the activation of alternative receptor tyrosine kinases, such as AXL and MET, which sustain PI3K/AKT signaling and bypass the dependency on EGFR [34,35].

Preclinical studies suggest that the miR-200c/LIN28B axis contributes to acquired resistance. In resistant cells, the miR-200c promoter undergoes hypermethylation, leading to its silencing. This, in turn, causes the upregulation of LIN28B, an RNA-binding protein that blocks the maturation of the let-7 family of miRNAs. The subsequent decrease in mature let-7 leads to increased expression of its targets, including RAS, MYC, and HMGA2, which collectively drive stemness and drug tolerance [35,36]. Critically, restoring miR-200c expression or knocking down LIN28B can resensitize cells to EGFR-TKIs in vitro, validating this pathway as a viable therapeutic target. Clinical studies have corroborated these findings, showing that circulating miR-200c levels decline in patients prior to radiographic progression on EGFR-TKI therapy, suggesting its potential utility for monitoring emergent resistance [37,38].

Interpretation and Clinical Implications: These findings suggest that while low miR-200 expression contributes to TKI resistance, its restoration alone may be insufficient due to co-occurring bypass pathway activation, such as MET or AXL. A more rational approach may involve dual-targeting strategies that combine miR-200 restoration or epigenetic reactivation with agents targeting the tumor microenvironment, like anti-VEGF therapy. Therefore, the critical next step is to design biomarker-stratified trials evaluating such combinations in patients with EMT-high tumors, using serial liquid biopsies to monitor response and confirm predictive value.

### 4.2. Immuno-Oncology

The miR-200/ZEB1 circuit directly controls Programmed Death-Ligand 1 (PD-L1) by regulating its transcription and targeting its 3′-UTR, thereby linking EMT to an immune-evasive phenotype [5]. Clinical validation for this axis comes from the work of Katakura et al., who demonstrated a significant inverse correlation between miR-200b and PD-L1 tumor proportion score (TPS) in both tumor cells and serum-derived exosomes from NSCLC patients. These findings were functionally corroborated in NSCLC cell lines, where miR-200b levels directly modulated PD-L1 expression. The data strongly support miR-200b as a minimally invasive surrogate biomarker for PD-L1, mechanistically connecting EMT-driven miRNA dysregulation to immune evasion [39].

The functional consequences of this regulation extend to the tumor immune microenvironment. Tumors characterized by low miR-200 and high ZEB1 expression exhibit reduced infiltration of CD8+ T cells, increased expression of T cell exhaustion markers (e.g., PD-1, TIM-3, LAG-3), and diminished cytotoxic effector function [40]. Mechanistically, ZEB1 upregulates immunosuppressive chemokines, such as CXCL1 and CXCL2, which recruit myeloid-derived suppressor cells, while simultaneously reducing signals that attract T cells [41]. This suggests a complex therapeutic implication: mesenchymal tumors with low miR-200 expression may, despite their poor prognosis, be preferentially responsive to PD-1/PD-L1 blockade due to high baseline PD-L1 levels. Conversely, epithelial tumors with high miR-200 expression may require alternative immune activation strategies to overcome their limited baseline immunogenicity [42].

Interpretation. While the inverse correlation between miR-200 and PD-L1 links EMT to immune evasion, this relationship is complex. PD-L1 expression is also dynamically induced by inflammatory signals like IFN-γ, potentially overriding miR-200-mediated repression. This interplay involves both cell-intrinsic PD-L1 derepression after miR-200 loss and cell-extrinsic mechanisms, where the EMT program reshapes the tumor microenvironment to suppress CD8+ T cell infiltration. Consequently, the predictive value of miR-200 for immune checkpoint inhibitor (ICI) therapy remains a hypothesis until its incremental benefit over established biomarkers like PD-L1 and TMB is clinically validated.

Because circuit-level regulation of PD-L1 and T cell infiltration is measurable in tissue and circulation, we proceed to evaluate miR-200 as a clinical biomarker across diagnostic, prognostic, and predictive settings.

**Figure 2:**
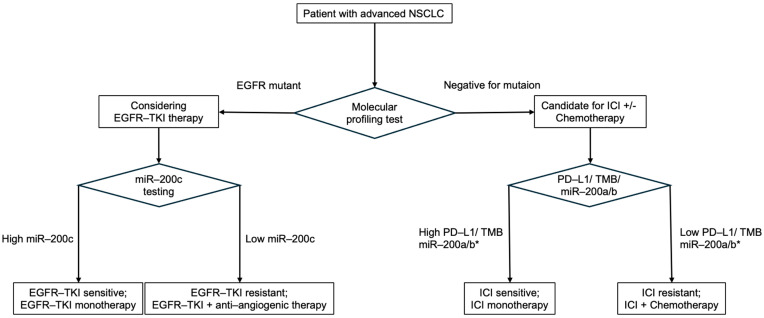
Proposed, hypothesis-generating algorithm for advanced NSCLC integrating miR-200 status with decision nodes. Patients are first stratified by oncogenic driver alterations. In driver-positive tumors (e.g., EGFR-mutant), targeted therapy with EGFR-TKIs is the standard, with anti-angiogenic combinations as an option; low miR-200 (EMT-high) may signal higher risk of tolerance and benefit from add-on strategies, whereas high miR-200 suggests TKI sensitivity. In driver-negative tumors, PD-L1 tumor proportion score (TPS) remains the primary determinant (≥50%: ICI monotherapy; 1–49% or <1%: chemo-ICI), with miR-200 considered as a potential modifier. * miR-200a/b: In NSCLC, low serum miR-200a is linked to high PD-L1 and better ICI response, while high miR-200 reflects EMT suppression that may also support ICI sensitivity. Their effect is context-dependent. Abbreviations: NSCLC, non-small-cell lung cancer; TKI, tyrosine kinase inhibitor; ICI, immune checkpoint inhibitor; PD-L1, programmed death-ligand 1; EMT, epithelial–mesenchymal transition; TMB, tumor mutational burden.

A hypothesis-generating clinical workflow that integrates miR-200 status with driver mutations and PD-L1 expression is summarized in Figure 2.

## 5. Clinical Utility of miR-200 as a Biomarker

The clinical utility of the miR-200 family as a biomarker in NSCLC is supported by a growing body of evidence across diagnostic, prognostic, and predictive contexts. Key diagnostic, prognostic, and predictive studies of the miR-200 family in NSCLC are summarized in Table 1.

Diagnostic applications have been explored using both tissue and liquid biopsy samples. A comprehensive meta-analysis reported a pooled sensitivity of 73% and specificity of 85% for NSCLC detection, yielding an area under the curve (AUC) of 0.83 [6]. Performance was superior in tissue specimens compared to blood, and quantitative RT-PCR demonstrated better reproducibility than microarray platforms.

Prognostic value is robustly established, with studies demonstrating that miR-200 expression is an independent predictor of overall survival in multivariate analyses that account for key clinical variables such as stage and histology [7].

Predictive applications are context-dependent. In the setting of EGFR-TKI therapy for EGFR-mutant patients, high miR-200c expression correlates with improved response rates and longer progression-free survival [8]. For immunotherapy, emerging clinical data suggest that serum miR-200 levels may predict outcomes, although the relationship is complex and requires further validation [9].

Liquid biopsy approaches, particularly those analyzing miRNAs within extracellular vesicles (EVs), are promising due to the enhanced stability and tumor-specificity of these miRNAs compared to their free circulating counterparts, which are found in various body fluids [43,44]. Recent studies have validated the diagnostic potential of EV-derived miR-200, showing high accuracy for detecting early-stage disease. However, widespread clinical adoption is hindered by a lack of standardization in pre-analytical and analytical variables.

Evidence Grading and Limitations. Although the miR-200 family shows promise as a biomarker, the strength of the current evidence is uneven across different clinical applications. Its prognostic value is the most consistently reported, whereas its diagnostic accuracy is moderate, and its predictive capacity is more established for EGFR-TKI response than for immunotherapy. However, widespread clinical adoption is hindered by major limitations, including small study sizes, lack of external validation, and significant heterogeneity in assay platforms and pre-analytical methods. Establishing standardized protocols and performing large-scale validation studies are therefore essential prerequisites for its integration into clinical practice.

Taken together, assayable miR-200 signatures hold actionable predictive value and enable longitudinal monitoring; we therefore survey therapeutic strategies that either restore miR-200 activity or exploit vulnerabilities of the EMT state.

**Table 1:** Cross-study summary of miR-200-based biomarkers in NSCLC.

Study (Year)	Design	Cohort Size (*n*)	Stage	Platform	Analyte	Endpoint	Effect Size/AUC (as Reported)
Ling & Yang (2024) [6]	Meta-analysis (diagnostic)	16 studies/20 cohorts	Mixed (LUAD/SCC; some SCLC)	Mixed (predominantly RT-qPCR)	Mixed (blood/tissue/sputum/LN)	Diagnostic	Pooled AUC 0.83, sensitivity 0.73, specificity 0.85
Liu (2024) [44]	Single-center retrospective (diagnostic)	NSCLC 168; benign 128; healthy 100 (Total 396)	Early-stage NSCLC	RT-qPCR (EV miRNA)	Plasma EVs	Diagnostic	AUC 0.855 (miR-200 alone); 0.942 (miR-200 + CA242/CEA/CA199)
Si (2017) [7]	Single-cohort prognostic (resected)	NSCLC 110 (43 healthy controls for expression comparison)	Post-resection NSCLC (I–III; details in text)	RT-qPCR (tissue)	Tumor tissue	OS/DFS	High miR-200c associated with poorer 5-yr DFS/OS (multivariable significant); no AUC
Tejero (2014) [27]	Single-cohort prognostic (resected, early)	Total 155 (Stage I subset *n* = 94; ADC *n* = 73, SCC *n* = 70)	Early-stage NSCLC (resected)	RT-qPCR (tissue)	Tumor tissue	OS	Risk score (miR-141 + miR-200c) independently associated with OS (OR 2.787; *p* = 0.033)
Li (2014) [8]	Single-cohort predictive (EGFR-TKI in advanced NSCLC)	Total 150 (EGFR WT subgroup *n* = 66)	Advanced NSCLC (2nd/3rd-line TKI)	RT-qPCR (tissue)	Tumor tissue	PFS/OS/DCR (on EGFR-TKIs)	In EGFR WT: higher miR-200c → ↑DCR, longer PFS/OS (text reports group differences)
Kaneko (2025) [9]	Prospective translational (ICI predictive)	Advanced NSCLC *n* = 16	IIIB-IVB	ddPCR (serum) + IHC (PD-L1)	Serum	PFS (ICI)/PD-L1 correlation	Low serum miR-200a → longer PFS (200 vs. 129 days; *p* = 0.008)
Katakura (2020) [39]	Translational correlation (PD-L1 surrogate)	*n* = 29	Mixed stages (I–IV)	RT-qPCR (cells/exosomes) + IHC (PD-L1)	Tissue & serum-EV	PD-L1 correlation	Negative correlations (e.g., r ≈ −0.36 to −0.37) between miR-200b and PD-L1

## 6. Therapeutic Strategies and Combinations

The central role of the miR-200/ZEB circuit in NSCLC progression makes it an attractive target for therapeutic intervention. Several strategies, overviewed in general reviews of miRNA therapeutics [45], are currently under investigation.

MiRNA Replacement Therapy: The most direct approach involves the use of synthetic miR-200 mimics. Preclinical studies using lipid nanoparticle formulations have shown tumor-selective delivery and sustained target engagement. Clinical translation will require optimization of dosing, delivery vehicles, and strategies to minimize off-target effects in normal epithelial tissues.

Clinical lessons: miRNA mimic trials have shown both therapeutic potential and significant safety concerns [46,47,48]. MRX34 (liposomal miR-34a) demonstrated target engagement and responses but was terminated after immune-mediated adverse events, including cytokine reactions and immune hepatitis, resulting in four treatment-related deaths. Similarly, TargomiRs (EGFR-targeted miR-16 mimic) showed activity but caused frequent infusion reactions and anaphylactoid events. These outcomes highlight risks from innate immune activation and delivery vehicle toxicity, emphasizing the need for improved safety strategies.

On-target/off-tumour considerations for the miR-200 family: The miR-200 family is physiologically enriched in epithelial tissues and enforces epithelial identity by antagonising EMT networks (e.g., ZEB1/2). While this epithelial “gate-keeping” is desirable in carcinoma cells, supraphysiologic replacement in normal epithelia could theoretically impede EMT-dependent re-epithelialisation during wound healing and mucosal repair [21,49]. Moreover, alveolar and airway epithelia express miR-200 family members and dynamically modulate them during injury and fibrosis, implying the need to avoid broad exposure in healthy epithelium.

Risk mitigation framework: To reduce innate immune activation and constrain on-target/off-tumour effects when developing miR-200 mimics, we propose: (i) chemistry that dampens TLR7/8 and other sensors (2′-O-methyl/2′-F/LNA bases and judicious phosphorothioate use) to reduce cytokine-driven reactions [50,51,52]; (ii) delivery vehicles that localise exposure—ligand-directed lipid nanoparticles (LNPs) (e.g., EGFR- or HER3-targeting, or optimised inhaled/nebulised LNPs for thoracic disease) [53], hepatocyte-specific GalNAc conjugation where confinement to liver is desired [54,55], and antibody-oligonucleotide conjugates (AOCs) that exploit tumour antigens [56]; and (iii) expression gating when using vectorised (expressed) miRNA systems—tumour-selective promoters (e.g., survivin/BIRC5, hTERT) and/or microRNA-response-element detargeting to silence expression in normal epithelia [57,58]. Collectively, these strategies aim to preserve anti-tumour efficacy while mitigating risk to epithelia that natively express miR-200.

Epigenetic Reactivation: This strategy aims to reverse the epigenetic silencing of miR-200 expression in mesenchymal tumors. DNA methyltransferase (DNMT) inhibitors (e.g., 5-azacytidine) and histone deacetylase (HDAC) inhibitors (e.g., vorinostat) have been shown to restore miR-200 expression, with synergistic effects observed when used in combination [59,60].

Downstream Targeting: While ZEB1 and ZEB2 are challenging to target directly, novel approaches such as proteolysis-targeting chimeras (PROTACs) offer new possibilities [61]. LIN28B represents a more druggable downstream node, and small-molecule inhibitors are in preclinical development for overcoming EGFR-TKI resistance [62,63,64].

Rational Combinations: Exploiting the circuit’s biology to create synthetic lethalities is a promising avenue. Combining EGFR-TKIs with miR-200 restoration has shown synergistic effects in organoid models [34]. Similarly, combining EMT reversal agents with anti-angiogenic therapies or with immune checkpoint inhibitors could simultaneously target tumor invasion, vascular supply, and immune recognition [65,66]. Finally, restoring an epithelial state can sensitize tumors to conventional chemotherapy by enhancing apoptotic sensitivity and reducing the activity of drug efflux pumps [67].

Translational Barriers and Development Pathway: Translating these therapeutic strategies into clinical practice faces significant barriers. For instance, miRNA replacement therapies must overcome challenges in targeted delivery, potential immunogenicity, and off-tumor effects, while epigenetic reactivation strategies raise concerns about non-specific toxicity. A pragmatic development path involves early-phase trials anchored by pharmacodynamic endpoints in patients selected for EMT-high signatures. These trials should test rational combinations, such as pairing EMT modulators with EGFR-TKIs or immune checkpoint inhibitors. Ultimately, demonstrating a clear clinical benefit over existing standards of care will be essential for regulatory approval.

These avenues motivate a translation roadmap that prioritises assay standardisation, biomarker-stratified trial designs, and drug discovery efforts against transcription factors—detailed in the subsequent Future Directions.

## 7. Future Perspectives and Unresolved Questions

In light of the above, we distil the principal clinical messages while avoiding repetition of the forthcoming future-facing priorities. Despite significant progress, several barriers must be overcome to translate our understanding of the miR-200 family into clinical practice.

### 7.1. Limitations and Caveats

Evidence synthesis is constrained by (i) spatiotemporal heterogeneity of EMT states and sampling discordance; (ii) variability in pre-analytical handling and miRNA quantification platforms; (iii) predominance of retrospective or cross-sectional designs with residual confounding; (iv) limited spatial and single-cell resolution linking miR-200 states to immune contexture [68]; and (v) uncertain generalizability across histologies and treatment lines. These limitations temper causal inferences and necessitate prospective, harmonized studies before clinical adoption [69].

### 7.2. Trial Design Priorities

We advocate biomarker-stratified, randomized designs that (a) pre-specify EMT-high/miR-200-low strata; (b) embed serial EV-miR-200 and spatial profiling for pharmacodynamics; (c) compare standard-of-care ± EMT-targeted add-ons (anti-VEGF, epigenetic priming, miR-200 mimic); (d) test incremental predictive value over PD-L1/TMB/IFN-γ scores using decision-curve analysis; and (e) adopt iRECIST/EMT-adapted endpoints for ICI-containing regimens. Falsifiable predictions (e.g., early EV-miR-200 rise → restored epithelial markers → improved TKI sensitivity) should be prospectively specified [70,71].

## 8. Conclusions

The miR-200/ZEB circuit integrates EMT, therapy tolerance, and immune contexture, positioning it as a central regulatory node with translational potential. Our synthesis highlights where evidence is strongest (prognostic tissue markers; EGFR-TKI contexts) and where it remains emergent (predicting ICI response; liquid-biopsy standardization). Near-term priorities are assay harmonization, spatial/longitudinal validation, and biomarker-stratified trials that demonstrate incremental clinical utility. Real-world implementation will likely require coordinated efforts across basic, translational, clinical, and regulatory domains.

## Data Availability

No new data were created or analyzed in this study.

## Abbreviations

The following abbreviations are used in this manuscript:

NSCLC	Non-small-cell lung cancer
miR-200	microRNA-200 family
ZEB1/2	Zinc finger E-box-binding homeobox 1/2
EMT	Epithelial–mesenchymal transition
EGFR	Epidermal growth factor receptor
PD-L1	Programmed death-ligand 1
CD8+ T cell	Cluster of differentiation 8-positive T lymphocyte
TKI	Tyrosine kinase inhibitor
TGF-β	Transforming growth factor beta
Wnt	Wingless/INT signaling pathway
LEF	Lymphoid enhancer-binding factor
HEY1/2	Hairy/enhancer-of-split related with YRPW motif 1/2
CpG	Cytosine-phosphate-guanine dinucleotide
VEGFR1	Vascular endothelial growth factor receptor 1
FLT1	Fms-related tyrosine kinase 1
CXCL	C-X-C motif chemokine ligand
CSC	Cancer stem cell
CD133	Cluster of differentiation 133
ALDH1	Aldehyde dehydrogenase 1
PI3K	Phosphoinositide 3-kinase
AKT	Protein kinase B
LIN28B	Lin-28 homolog B
3′-UTR	3′ untranslated region
TPS	Tumor proportion score
PD-1	Programmed cell death protein 1
TIM-3	T cell immunoglobulin and mucin domain-containing protein 3
LAG-3	Lymphocyte activation gene 3
AUC	Area under the curve
RT-PCR	Reverse-transcription polymerase chain reaction
EV	Extracellular vesicle
DNMT	DNA methyltransferase
HDAC	Histone deacetylase

## References

[1] Zhou J., Xu Y., Liu J., Feng L., Yu J., Chen D. (2024). Global Burden of Lung Cancer in 2022 and Projections to 2050: Incidence and Mortality Estimates from GLOBOCAN. Cancer Epidemiol..

[2] Gregory P.A., Bert A.G., Paterson E.L. (2008). The MiR-200 Family and MiR-205 Regulate Epithelial to Mesenchymal Transition by Targeting ZEB1 and SIP1. Nat. Cell Biol..

[3] Park S.M., Gaur A.B., Lengyel E., Peter M.E. (2008). The MiR-200 Family Determines the Epithelial Phenotype of Cancer Cells by Targeting the E-Cadherin Repressors ZEB1 and ZEB2. Genes Dev..

[4] Sato H., Shien K., Tomida S. (2017). Targeting the MiR-200c/LIN28B Axis in Acquired EGFR-TKI Resistance Non-Small Cell Lung Cancer Cells Harboring EMT Features. Sci. Rep..

[5] Chen L., Gibbons D.L., Goswami S., Cortez M.A., Ahn Y.H., Byers L.A., Zhang X., Yi X., Dwyer D., Lin W. (2014). Metastasis Is Regulated via MicroRNA-200/ZEB1 Axis Control of Tumour Cell PD-L1 Expression and Intratumoral Immunosuppression. Nat. Commun..

[6] Ling Z., Yang L. (2024). The Diagnostic Value of Circulating MiR-200 Family in Non–Small Cell Lung Cancer: An Updated Meta-Analysis. Biomark. Med..

[7] Si L., Tian H., Yue W., Li L., Li S., Gao C., Qi L. (2017). Potential use of microRNA-200c as a prognostic marker in non-small cell lung cancer. Oncol. Lett..

[8] Li J., Li X., Ren S., Chen X., Zhang Y., Zhou F., Zhao M., Zhao C., Chen X., Cheng N. (2014). miR-200c overexpression is associated with better efficacy of EGFR-TKIs in non-small cell lung cancer patients with EGFR wild-type. Oncotarget.

[9] Kaneko A., Kobayashi N., Kubo S., Nagaoka S., Muraoka S., Fukuda N., Somekawa K., Matsumoto H., Katakura S., Teranishi S. (2025). MiR-200a Regulates PD-L1 and Predicts Response to Immune Checkpoint Inhibitors in Advanced Non-Small Cell Lung Cancer. Transl. Lung Cancer Res..

[10] Kobayashi N., Miura K., Kaneko A., Matsumoto H., Somekawa K., Hirose T. (2023). Tailoring Therapeutic Strategies in Non-Small-Cell Lung Cancer: The Role of Genetic Mutations and Programmed Death Ligand-1 Expression in Survival Outcomes. Cancers.

[11] Reck M., Remon J., Hellmann M.D. (2022). First-Line Immunotherapy for Non-Small-Cell Lung Cancer. J. Clin. Oncol..

[12] Bracken C.P., Gregory P.A., Kolesnikoff N. (2008). A Double-Negative Feedback Loop between ZEB1-SIP1 and the MicroRNA-200 Family Regulates Epithelial-Mesenchymal Transition. Cancer Res..

[13] Korpal M., Lee E.S., Hu G., Kang Y. (2008). The MiR-200 Family Inhibits Epithelial-Mesenchymal Transition and Cancer Cell Migration by Direct Targeting of E-Cadherin Transcriptional Repressors ZEB1 and ZEB2. J. Biol. Chem..

[14] Burk U., Schubert J., Wellner U. (2008). A Reciprocal Repression between ZEB1 and Members of the MiR-200 Family Promotes EMT and Invasion in Cancer Cells. EMBO Rep..

[15] Brabletz S., Brabletz T. (2010). The ZEB/MiR-200 Feedback Loop—A Motor of Cellular Plasticity in Development and Cancer?. EMBO Rep..

[16] Watanabe K., Panchy N., Noguchi S. (2019). Combinatorial Perturbation Analysis Reveals Divergent Regulations of the Same Transcription Factor Target Genes. PLoS Genet..

[17] Roca H., Hernandez J., Weidner S. (2013). Transcription Factors OVOL1 and OVOL2 Induce the Mesenchymal to Epithelial Transition in Human Cancer. PLoS ONE.

[18] Lamouille S., Xu J., Derynck R. (2014). Molecular Mechanisms of Epithelial-Mesenchymal Transition. Nat. Rev. Mol. Cell Biol..

[19] Nieto M.A., Huang R.Y., Jackson R.A., Thiery J.P. (2016). EMT: 2016. Cell.

[20] Davalos V., Moutinho C., Villanueva A. (2012). Dynamic Epigenetic Regulation of the MicroRNA-200 Family Mediates Epithelial and Mesenchymal Transitions in Human Tumorigenesis. Oncogene.

[21] Cavallari I., Ciccarese F., Sharova E. (2021). The MiR-200 Family of MicroRNAs: Fine Tuners of Epithelial-Mesenchymal Transition and Circulating Cancer Biomarkers. Cancers.

[22] Gibbons D.L., Lin W., Creighton C.J. (2009). Contextual Extracellular Cues Promote Tumor Cell EMT and Metastasis by Regulating MiR-200 Family Expression. Genes Dev..

[23] Kobayashi N., Katakura S., Fukuda N., Somekawa K., Kaneko A., Kaneko T. (2024). The Impact of Bevacizumab and MiR–200c on EMT and EGFR–TKI Resistance in EGFR–Mutant Lung Cancer Organoids. Genes.

[24] Wellner U., Schubert J., Burk U.C. (2009). The EMT-Activator ZEB1 Promotes Tumorigenicity by Repressing Stemness-Inhibiting MicroRNAs. Nat. Cell Biol..

[25] Ahmad A. (2013). Pathways to Breast Cancer Recurrence. ISRN Oncol..

[26] Ceppi P., Mudduluru G., Kumarswamy R. (2010). Loss of MiR-200c Expression Induces an Aggressive, Invasive, and Chemoresistant Phenotype in Non-Small Cell Lung Cancer. Mol. Cancer Res..

[27] Tejero R., Navarro A., Campayo M. (2014). MiR-141 and MiR-200c as Markers of Overall Survival in Early Stage Non-Small Cell Lung Cancer Adenocarcinoma. PLoS ONE.

[28] Puram S.V., Tirosh I., Parikh A.S. (2017). Single-Cell Transcriptomic Analysis of Primary and Metastatic Tumor Ecosystems in Head and Neck Cancer. Cell.

[29] Pastushenko I., Brisebarre A., Sifrim A. (2018). Identification of the Tumour Transition States Occurring during EMT. Nature.

[30] Mani S.A., Guo W., Liao M.J. (2008). The Epithelial-Mesenchymal Transition Generates Cells with Properties of Stem Cells. Cell.

[31] Morel A.P., Lièvre M., Thomas C. (2008). Generation of Breast Cancer Stem Cells through Epithelial-Mesenchymal Transition. PLoS ONE.

[32] Ye X., Tam W.L., Shibue T. (2015). Distinct EMT Programs Control Normal Mammary Stem Cells and Tumour-Initiating Cells. Nature.

[33] Zhang Z., Lee J.C., Lin L. (2012). Activation of the AXL Kinase Causes Resistance to EGFR-Targeted Therapy in Lung Cancer. Nat. Genet..

[34] Sequist L.V., Waltman B.A., Dias-Santagata D., Digumarthy S., Turke A.B., Fidias P., Bergethon K., Shaw A.T., Gettinger S., Cosper A.K. (2011). Genotypic and Histological Evolution of Lung Cancers Acquiring Resistance to EGFR Inhibitors. Sci. Transl. Med..

[35] Engelman J.A., Zejnullahu K., Mitsudomi T. (2007). MET Amplification Leads to Gefitinib Resistance in Lung Cancer by Activating ERBB3 Signaling. Science.

[36] Viswanathan S.R., Daley G.Q., Gregory R.I. (2008). Selective Blockade of MicroRNA Processing by Lin28. Science.

[37] Wang H.Y., Liu S.G., Wu S.G. (2020). MiR-200c-3p Suppression Is Associated with Development of Acquired Resistance to Epidermal Growth Factor Receptor (EGFR) Tyrosine Kinase Inhibitors in EGFR Mutant Non-Small Cell Lung Cancer via a Mediating Mechanism of Epithelial-Mesenchymal Transition. Cancer Biomark..

[38] Zhou G., Zhang F., Guo Y., Huang J., Xie Y., Yue S., Chen M., Jiang H., Li M. (2017). MiR–200c Enhances Sensitivity of Drug–Resistant Non–Small Cell Lung Cancer to Gefitinib by Suppression of PI3K/Akt Signaling Pathway and Inhibits Cell Migration via Targeting ZEB1. Biomed. Pharmacother..

[39] Katakura S., Kobayashi N., Hashimoto H. (2020). MicroRNA-200b Is a Potential Biomarker of the Expression of PD-L1 in Patients with Lung Cancer. Thorac. Cancer.

[40] Thommen D.S., Schumacher T.N. (2018). T Cell Dysfunction in Cancer. Cancer Cell.

[41] Kudo-Saito C., Shirako H., Takeuchi T., Kawakami Y. (2009). Cancer Metastasis Is Accelerated through Immunosuppression during Snail-Induced EMT of Cancer Cells. Cancer Cell.

[42] Herbst R.S., Morgensztern D., Boshoff C. (2018). The Biology and Management of Non-Small Cell Lung Cancer. Nature.

[43] Weber J.A., Baxter D.H., Zhang S. (2010). The MicroRNA Spectrum in 12 Body Fluids. Clin. Chem..

[44] Liu Q. (2024). Diagnostic Value of Extracellular Vesicle-Derived MiR-200 Family Members for Non-Small Cell Lung Cancer. Clin. Exp. Med..

[45] Rupaimoole R., Slack F.J. (2017). MicroRNA Therapeutics: Towards a New Era for the Management of Cancer and Other Diseases. Nat. Rev. Drug Discov..

[46] Beg M.S., Brenner A.J., Sachdev J., Borad M., Kang Y.-K., Stoudemire J., Smith S., Bader A.G., Kim S. (2017). Phase I Study of MRX34, a Liposomal MiR-34a Mimic. Investig. New Drugs.

[47] Hong D.S., Kang Y.-K., Borad M., Sachdev J., Ejadi S., Lim H.Y., Brenner A.J., Park K., Lee J.-L., Kim T.-Y. (2020). Phase 1 Study of MRX34, a Liposomal MiR-34a Mimic, in Patients with Advanced Solid Tumours. Br. J. Cancer.

[48] van Zandwijk N., Pavlakis N., Kao S.C., Linton A., Boyer M.J., Clarke S., Huynh Y., Chrzanowska A., Fulham M.J., Bailey D.L. (2017). Safety and Activity of MicroRNA-Loaded Minicells in Patients with Recurrent Malignant Pleural Mesothelioma: A First-in-Man, Phase 1, Open-Label, Dose-Escalation Study. Lancet Oncol..

[49] Tang H., Wang X., Zhang M., Yan Y., Huang S., Ji J., Xu J., Zhang Y., Cai Y., Yang B. (2020). MicroRNA-200b/c-3p Regulate Epithelial Plasticity and Inhibit Cutaneous Wound Healing by Modulating TGF-β-Mediated RAC1 Signaling. Cell Death Dis..

[50] Kanasty R.L., Whitehead K.A., Vegas A.J., Anderson D.G. (2012). Action and reaction: The biological response to siRNA and its delivery vehicles. Mol. Ther..

[51] Jung S., von Thülen T., Laukemper V., Pigisch S., Hangel D., Wagner H., Kaufmann A., Bauer S. (2015). A Single Naturally Occurring 2′-O-Methylation Converts a TLR7- and TLR8-Activating RNA into a TLR8-Specific Ligand. PLoS ONE.

[52] Seyhan A.A. (2024). Trials and Tribulations of MicroRNA Therapeutics. Int. J. Mol. Sci..

[53] Leong E.W.X., Ge R. (2022). Lipid Nanoparticles as Delivery Vehicles for Inhaled Therapeutics. Biomedicines.

[54] Springer A.D., Dowdy S.F. (2018). GalNAc-siRNA Conjugates: Leading the Way for Delivery of RNAi Therapeutics. Nucleic Acid Ther..

[55] An G. (2024). Pharmacokinetics and Pharmacodynamics of GalNAc–Conjugated SiRNAs. J. Clin. Pharmacol..

[56] Malecova B., Burke R.S., Cochran M., Hood M.D., Johns R., Kovach P.R., Doppalapudi V.R., Erdogan G., Arias J.D., Darimont B. (2023). Targeted tissue delivery of RNA therapeutics using antibody-oligonucleotide conjugates (AOCs). Nucleic Acids Res..

[57] Montaño-Samaniego M., Bravo-Estupiñan D.M., Méndez-Guerrero O., Alarcón-Hernández E., Ibáñez-Hernández M. (2020). Strategies for Targeting Gene Therapy in Cancer Cells With Tumor-Specific Promoters. Front. Oncol..

[58] Merlin S., Follenzi A. (2019). Transcriptional Targeting and MicroRNA Regulation of Lentiviral Vectors. Mol. Ther. Methods Clin. Dev..

[59] Zhang Y., Wang J., Chen G. (2017). Inhibition of Hypermethylation Restores MiR-200c in Hepatocellular Carcinoma by Targeting ZEB1. Int. J. Oncol..

[60] Ning X., Shi Z., Liu X. (2015). DNMT1 and EZH2 Mediated Methylation Silences the MicroRNA-200b/a/429 Gene and Promotes Tumor Progression. Cancer Lett..

[61] Jung H., Lee Y. (2025). Targeting the Undruggable: Recent Progress in PROTAC–Induced Transcription Factor Degradation. Cancers.

[62] Roos M., Pradere U., Ngondo R.P., Behera A., Allegrini S., Civenni G., Zagalak J.A., Marchand J.-R., Menzi M., Towbin H. (2016). A Small–Molecule Inhibitor of Lin28. ACS Chem. Biol..

[63] Wang L., Rowe R.G., Jaimes A., Yu C., Nam Y., Pearson D.S., Zhang J., Xie X., Marion W., Heffron G.J. (2018). Small–Molecule Inhibitors Disrupt Let–7 Oligouridylation and Release the Selective Blockade of Let–7 Processing by LIN28. Cell Rep..

[64] Borgelt L., Li F., Hommen P., Lampe P., Hwang J., Goebel G.L., Sievers S., Wu P. (2021). Trisubstituted Pyrrolinones as Small–Molecule Inhibitors Disrupting the Protein–RNA Interaction of LIN28 and Let–7. ACS Med. Chem. Lett..

[65] Qian W., Zhao M., Wang R., Li H. (2021). Fibrinogen-like protein 1 (FGL1): The next immune checkpoint target. J. Hematol. Oncol..

[66] Lou Y., Diao L., Cuentas E.R. (2016). Epithelial-Mesenchymal Transition Is Associated with a Distinct Tumor Microenvironment Including Elevation of Inflammatory Signals and Multiple Immune Checkpoints in Lung Adenocarcinoma. Clin. Cancer Res..

[67] Fischer K.R., Durrans A., Lee S. (2015). Epithelial-to-Mesenchymal Transition Is Not Required for Lung Metastasis but Contributes to Chemoresistance. Nature.

[68] Lawson D.A., Bhakta N.R., Kessenbrock K. (2015). Single-Cell Analysis Reveals a Stem-Cell Program in Human Metastatic Breast Cancer Cells. Nature.

[69] Mitchell P.S., Parkin R.K., Kroh E.M., Fritz B.R., Wyman S.K., Pogosova-Agadjanyan E.L., Peterson A., Noteboom J., O’Briant K.C., Allen A. (2008). Circulating MicroRNAs as Stable Blood-based Markers for Cancer Detection. Proc. Natl. Acad. Sci. USA.

[70] Seymour L., Bogaerts J., Perrone A. (2017). IRECIST: Guidelines for Response Criteria for Use in Trials Testing Immunotherapeutics. Lancet Oncol..

[71] Simon R. (2011). Genomic biomarkers in predictive medicine: An interim analysis. EMBO Mol. Med..

